# Comparison of the effects of choline alphoscerate and citicoline in patients with dementia disorders: a systematic review and meta-analysis

**DOI:** 10.3389/fneur.2025.1649661

**Published:** 2025-12-05

**Authors:** Getu Gamo Sagaro, Francesco Amenta

**Affiliations:** 1 School of Medicinal and Health Product Sciences, University of Camerino, Camerino, Italy; 2 School of Public Health, College of Health Sciences and Medicine, Wolaita Sodo University, Sodo, Ethiopia; 3 Research Department, International Radio Medical Center (C.I.R.M.), Rome, Italy

**Keywords:** dementia disorders, Alzheimer’s disease, choline alphoscerate, citicoline, cognitive dysfunction, memory function

## Abstract

**Background:**

Over 44 million people worldwide live with dementia, affecting their quality of life and well-being. Choline alphoscerate and citicoline supplements are commonly used to improve cognitive function in dementia patients. However, their efficacy remains inconsistent.

**Objective:**

This systematic review aimed to investigate and compare the effects of choline alphoscerate and citicoline on cognitive impairments, behavioural symptoms, and other clinical conditions in patients with dementia disorders.

**Methods:**

PubMed and Scopus were searched to identify relevant studies. We calculated weighted mean differences (WMD) or standardized mean differences (SMD) and 95% confidence intervals (CI) for continuous outcomes and odds ratios (OR) and 95% CI for binary outcomes.

**Results:**

This review included data from 358 participants across three randomized controlled trials (RCTs). As measured by the Sandoz Clinical Assessment for Geriatric Patients (SCAG), choline alphoscerate significantly improved clinical conditions in patients with dementia disorders compared with citicoline at the end of treatment [WMD: −3.92 (95% CI: −7.41 to −0.42)]. Specifically, our pooled analysis revealed that choline alphoscerate showed significant improvements in cognitive function, interpersonal relationships, affective disorders, apathy, and somatic functioning compared to citicoline at the end of treatment, as measured by the SCAG. However, there was no significant difference between the choline alphoscerate and citicoline treatment groups on memory or word fluency tests (WFT). Dropout rates for choline alphoscerate and citicoline were 9.4 and 6.7%, respectively [OR: 1.44 (95% CI: 0.66 to 3.13)], indicating no significant differences in acceptability.

**Conclusion:**

Our findings indicate that choline alphoscerate is more effective than citicoline in improving the clinical conditions of dementia patients.

**Systematic review registration:**

https://www.crd.york.ac.uk/PROSPERO/view/CRD42024626782, Identifier: CRD42024626782.

## 1 Introduction

Dementia is a condition linked to various neurodegenerative diseases, marked by a significant decline in cognitive abilities that affects daily activities (1). It notably impacts the aging population’s quality of life and well-being worldwide. Alzheimer’s disease (AD)-related dementia is characterized by a severe decline in cognitive function, leading to a loss of independence (2). Dementia affects not only older adults but also their families and public programs, with wide-ranging direct and indirect effects (3). In 2013, an estimated 44.35 million people worldwide were living with dementia, with projections reaching 75.62 million by 2030 and 135.46 million by 2050 (4). The economic impact of dementia is estimated at $600 billion globally (5). Because the incidence of dementia sharply increases after age 75, and with the projected growth in the elderly population, dementia cases are expected to triple by 2050 without new interventions to prevent or slow cognitive decline (6).

The treatment of dementia disorders remains a global challenge. Despite numerous clinical trials, no cure for dementia has been discovered (7). The optimal treatment to prevent or delay mild cognitive impairment (MCI) or dementia has not been identified yet (8). Various pharmacological and nonpharmacological interventions have been introduced, but the evidence regarding their efficacy, safety, and tolerability is conflicting and inconsistent. Pharmacological therapy is the primary intervention used to enhance clinical conditions or slow the progression of dementia syndromes (8). Nevertheless, studies on the efficacy of cholinesterase inhibitors (ChE-Is) have concluded that the class of compounds does not have a positive impact on the treatment of cognitive impairment caused by dementia (9–12). This could be attributed to a decline in the efficacy of ChE-Is treatment over time, and to the difficulty in treating specific patient categories such as older age groups, or patients with the concomitant presence of conditions such as bradycardia, bronchial asthma, or chronic obstructive pulmonary disease due to the treatment-associated adverse effects (13, 14).

Cholinergic precursors were the first approach to addressing cognitive impairment in adult-onset dementia disorders (15). Preclinical data indicate that cholinergic precursors can elevate acetylcholine levels in specific brain regions, potentially enhancing cholinergic neurotransmission under certain conditions (16). Choline alphoscerate and citicoline are clinically tested cholinergic precursors that have demonstrated efficacy, safety, and tolerability in patients with dementia and other neurological conditions (17, 18). Choline alphoscerate [L-αlpha-Glycero-phosphorylcholine (alpha-GPC), alpha-glycero-phosphocholine, choline alfoscerate, and SN-glycero-3-phosphocholine] is a precursor for acetylcholine synthesis, a neurotransmitter involved in various neurological processes (15, 18). It has demonstrated the ability to cross the blood–brain barrier (BBB) and reach the brain, making it a valuable compound for enhancing cognitive function and supporting brain health (19). Several clinical studies have highlighted its potential as a promising treatment for improving cognitive function, including thinking, orientation, language comprehension, learning abilities, and memory in patients with dementia and other neurological disorders (17, 20–22). On the other hand, citicoline (CDP-choline, cytidine 5′-diphosphocholine, and cytidine diphosphate-choline) is mainly known as a precursor for synthesizing phospholipids, particularly phosphatidylcholine (PC) (23). It activates the biosynthesis of structural phospholipids in neuronal membranes, enhances cerebral metabolism, and influences various neurotransmitter levels (23–25). Citicoline has been shown in multiple clinical trials to improve cognitive deficits in patients with dementia and other neurological conditions (26–29) through a mechanism related to immunogenic and/or neurotrophic effects in the microvascular niche. This leads to improvements in cognitive abilities, particularly memory and reaction time. However, in the large COBRIT trial, citicoline did not show improvement in functional or cognitive status in patients with traumatic brain injury (TBI) (30). Other comparative clinical trials also revealed that alpha-GPC may be a more effective treatment for improving cognitive function in individuals with mild to moderate multi-infarct dementia than citicoline (31, 32).

In summary, most clinical trials have shown that both alpha-GPC and citicoline can improve cognitive deficits in patients with dementia and other neurological conditions. However, some trials suggested that alpha-GPC is more effective than citicoline in improving cognition in patients with dementia disorders.

This systematic review and meta-analysis aimed to evaluate and compare the effects of alpha-GPC and citicoline on cognitive impairments, behavioural symptoms, and other clinical conditions in patients with dementia disorders. The findings of this study will assist healthcare providers, caregivers, and patients in making informed decisions about the most effective interventions for dementia disorders.

## 2 Methods

We published this systematic review protocol on PROSPERO in November 2024, with the registration number CRD42024626782. The systematic review was conducted according to the Preferred Items for Systematic Review and Meta-analysis (PRISMA) reporting standards (33).

### 2.1 Search strategy

We conducted a comprehensive systematic search in internationally recognized databases, specifically PubMed and Scopus, to gather studies that reported on memory functions, behavioural outcomes, and overall clinical conditions, including cognitive dysfunction, interpersonal relationships, affective disorders, apathy, and somatic dysfunction status of patients diagnosed with dementia disorders. This search included studies published up until October 2024. Further, the reference lists of the retrieved publications and review articles were reviewed. The key terms illustrated in Table 1 were used for searching PubMed and Scopus (see search strategy in Supplementary Table 1). Search terms were combined using Boolean operations, such as “AND” and “OR,” and the search was restricted to humans only. To accommodate variations in word forms, keep terms together, and combine concepts, we applied various search techniques to the databases, incorporating quotation marks, parentheses, and other truncation symbols along with the key terms. With this method, we ensured that we would not miss any articles that contained different word endings or forms of the search terms.

**Table 1:** Characteristics of the included trials.

Features	Study		
	Frattola et al. (41)	Muratorio et al. (31)	Di Perri et al. (32)
Sample size, *n*	126	112	120
Patients completed the study	117	97	115
Treatment group (TG), *n*	59	48	56
Comparator group (CG), *n*	58	49	59
Average age (in years) of TG	69.5 ± 8.2 (SD)	68.6 ± 6.5 (SD)	70.9 ± 0.7 (SE)
Average age (in years) of CG	68.6 ± 7.6 (SD)	68.5 ± 6.3 (SD)	69.9 ± 0.9 (SE)
Sex: male to female ratio	2:1	2.2:1	1.5:1
Study design	RCT	RCT	RCT
Treatment duration	90 days	90 days	90 days
SCAG score of TG at baseline, mean ± SD	52.07 ± 10.74	43.44 ± 13.65	60.9 ± 1.5
SCAG score of CG at baseline, mean ± SD	53.22 ± 10.40	43.08 ± 14.41	64.2 ± 1.3
WMS score of TG at baseline, mean ± SD	4.50 ± 2.85	72.00 ± 10.38	3.6 ± 0.4
WMS score of CG at baseline, mean ± SD	3.84 ± 2.50	71.54 ± 11.60	3.0 ± 0.3
WFT score of TG at baseline, mean ± SD	12.46 ± 6.87	7.56 ± 6.63	8.1 ± 0.9
WFT score of CG at baseline, mean ± SD	11.33 ± 5.43	7.93 ± 5.52	6.9 ± 0.7

### 2.2 Eligibility criteria and selection of studies

Studies were included if they met the following inclusion criteria: (1) Randomized controlled trials (RCTs); (2) Studies of participants over the age of 50 who have been diagnosed with dementia disorders; (3) Original articles reporting choline alphoscerate versus citicoline effects on dementia patients; (4) Trials assessing the clinical conditions of patients using various psychological assessment scales, including Sandoz Clinical Assessment for Geriatric Patients (SCAG), the memory logic test of the Wechsler Memory Scale (WMS), the Parkside Behaviour Rating Scale Modified (PBRSM), and the Word Fluency test (WFT), among others; (5) Trials reporting memory function, stimulated behaviour, and overall clinical conditions of patients across domains such as cognitive dysfunction, interpersonal relationships, affective disorders, apathy, and somatic dysfunctions; (6) Trials reporting dropout rates and adverse effects associated with treatments during the study periods; (7) Trials measuring continuous outcomes at the end of treatment (endpoint) and reporting as mean and standard deviation or standard error; (8) Full-text studies written in English and published in peer-reviewed journals; (9) Studies conducted on humans. Articles were excluded if they were: (1) Systematic reviews, narrative reviews, or meta-analyses; (2) Published in languages other than English; (3) Published only as abstracts or conference proceedings.

Two reviewers (GS and FA) independently screened titles and abstracts of all identified studies based on the inclusion and exclusion criteria. Both reviewers meticulously examined pertinent articles during the literature search and study selection process. Disagreements were resolved through discussion and consensus.

### 2.3 Data extraction

Data were extracted from the included trials using a standard extraction sheet. For continuous outcomes, the mean value of the outcome measurements in each group at the end of treatment, the standard deviation (SD) or standard error (SE), and the number of participants for whom the outcome was measured in each group were extracted from the included studies. For dichotomous outcomes, the number of patients who discontinued treatment and the number of adverse events during the treatment period for each group were extracted. Additionally, the first author’s name, the study year, the duration of the treatment, and each outcome measurement’s baseline and endpoint were extracted.

### 2.4 Risk bias assessment in included studies

The Cochrane Collaboration tool for randomized controlled trials (RCTs) was used to assess the methodological quality of selected studies (34). The selected RCTs were evaluated based on six domains, including the method of randomization, concealment of allocation, blinding of investigators and patients, blinding of outcome assessment, incomplete outcome data, selective outcome reporting, and other sources of bias. For each of the domains, a score of “yes,” “no” or “unclear” was assigned. Studies were classified based on the quality assessment into three categories: low-risk bias (all quality criteria met), moderate-risk bias (one or more quality criteria only partly met), and high-risk bias (one or more quality criteria not met).

### 2.5 Outcome measures

The primary outcomes were the overall patients’ clinical condition measured by SCAG global scores and SCAG evaluation of cognitive function, interpersonal relationships, affective disorders, apathy, and somatic functioning. The secondary outcomes were memory function measured by WMS, stimulated behaviour measured by WFT, and the acceptability and tolerability of alpha-GPC and citicoline.

### 2.6 Statistical analysis

The data analysis was conducted using the R programming language (Version 4.4.1) (35). The meta-analysis used the *metafor* package of R software (36). The post-treatment mean (at the end of treatment) was considered to determine the mean difference. Pooled analysis required the endpoint’s mean value, standard deviation (SD), and number of participants. For studies reporting only SE, it was converted into SD. When combining continuous data in meta-analysis, a weighted mean difference (WMD) and 95% confidence interval (CI) were calculated when the outcome measures in all studies were made on the same scale. However, if the studies assessed the same outcome but measured it on different scales, the standardized mean difference (SMD) and 95% CI were calculated (37, 38). We calculated the odds ratio (OR) with 95% CI for binary outcomes, particularly acceptability and tolerability. We assessed the heterogeneity of the meta-analysis using the *p*-value of chi-square (*χ*^2^ or chi^2^) statistics, considering it significant if the *p*-value was <0.1 (34). The level of heterogeneity was estimated by the *I*^2^ statistics, which indicates the percentage ratio of variability in effect estimates caused by heterogeneity rather than chance (34). The heterogeneity degree was considered unimportant, moderate, substantial, and considerable based on *I*^2^ values of 0–40%, 30–60%, 50–90%, and 75–100%, respectively (34). When statistical heterogeneity was absent or of low or moderate significance, a meta-analysis was performed for a continuous outcome using a fixed effect model with the inverse variance approach. On the other hand, when statistical heterogeneity with substantial significance was observed, a meta-analysis was conducted for a continuous outcome using a random effects model with the inverse variance method (39, 40).

## 3 Results

### 3.1 Included studies

**Figure 1:**
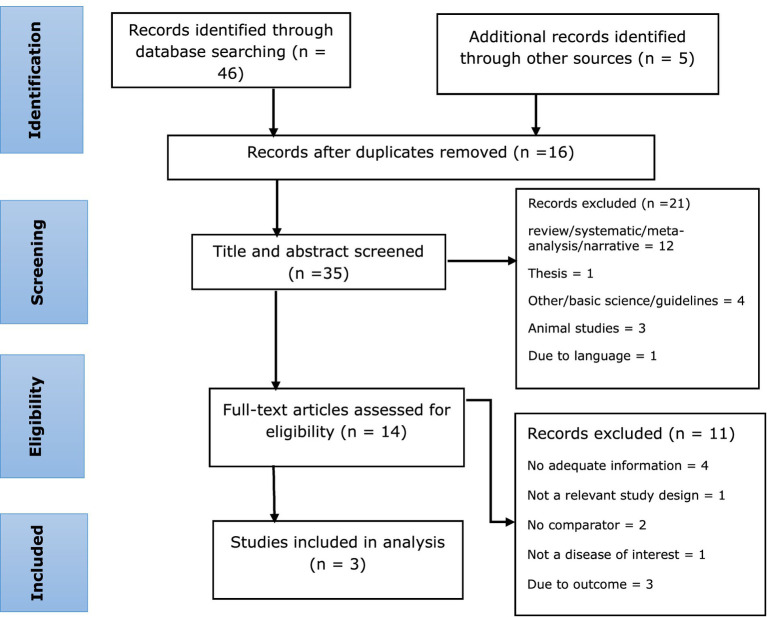
PRISMA flow chart for selecting eligible studies.

In conducting our literature search, we identified a total of 51 records. After a thorough review, we found that 16 records were duplicates and therefore excluded from further evaluation. Moving forward, we conducted a title and abstract screening of 35 studies, which excluded 21 additional studies. Following this initial screening process, we were left with 14 full-text articles that could be included in our study. However, upon careful examination of these articles, we determined that 11 of them did not meet our eligibility criteria and were subsequently excluded. After the screening process, we identified a total of three randomized controlled trials (RCTs) that met our eligibility criteria (31, 32, 41) (Figure 1).

### 3.2 Characteristics of the included trials

As presented in Table 1, both Frattola et al. (41) and Muratorio et al. (31) trials included patients who were affected with multi-infarct dementia (MID). On the other hand, Di Perri et al. (32) evaluated patients who were diagnosed with vascular dementia disorders. Frattola et al. (41) included 126 patients in their study. Of these, 117 patients completed the study. Among the 117 patients, 59 were treated with alpha-GPC while 58 were treated with citicoline. Muratorio et al. (31) trial involved 112 patients, with 57 individuals randomly assigned to receive alpha-GPC and 55 individuals receiving citicoline. However, 97 patients (48 in the alpha-GPC group and 49 in the citicoline group) completed the study. Di Perri et al. (32) included 120 patients in their study, all of whom were diagnosed with vascular dementia. Out of these, 60 patients were randomly assigned to receive alpha-GPC treatment, while the remaining 60 patients were allocated to the citicoline treatment group. Nevertheless, 115 patients out of 120 (56 patients received alpha-GPC and 59 patients were treated with citicoline) completed the study. A total of three RCTs involving 358 patients with dementia disorders were included in this systematic review. Three hundred and twenty-nine patients (92%) completed the study, of which 163 were in the alpha-GPC group and 166 were in the citicoline group. The patients received the treatment for 90 days in all the included studies. Frattola et al. (41) used the Sandoz Clinical Assessment for Geriatric Patients (SCAG) global scores to assess overall clinical conditions and their clusters such as cognitive dysfunction, interpersonal relationships, affective disorders, apathy, and somatic dysfunction; the memory logic test of the Wechsler Memory Scale (WMS) to evaluate memory function; and the Word Fluency test (WFT) to examine stimulated behaviour over 3 months. On the other hand, Muratorio et al. (31) utilized the SCAG, WMS, the Blessed Dementia Scale, Memory concentration test, the Rapid Disability Rating Scale 2 (RDRS 2), the Word Fluency Test, the token test, and the simple drawing copy (SDC) to assess the same parameters.

In Frattola et al. (41), the average age in years of the treatment and comparator groups was 69.5 ± 8.2 years and 68.6 ± 7.6 years, respectively. According to Muratorio et al. (31), the mean age of the treatment group was 68.6 ± 6.5 years, and that of the comparator group was 68.5 ± 6.3 years (Table 1). In the three trials, alpha-GPC and citicoline were administered intramuscularly at a dose of 1,000 mg per day for three months. Two of the included trials (32, 41) reported that alpha-GPC was significantly more effective than citicoline in improving patients with dementia across all clinical conditions, as measured by the SCAG global score and its domains, including cognitive function, interpersonal relationships, and somatic functioning. Two studies (31, 41) indicate that alpha-GPC has a significantly greater impact on improving memory functions, as measured by WMS, and stimulated behaviour, as measured by WFT, compared to citicoline.

### 3.3 Alpha-GPC versus citicoline effects on cognitive dysfunction, interpersonal relationships, affective disorders, apathy, and somatic dysfunction

**Figure 2:**
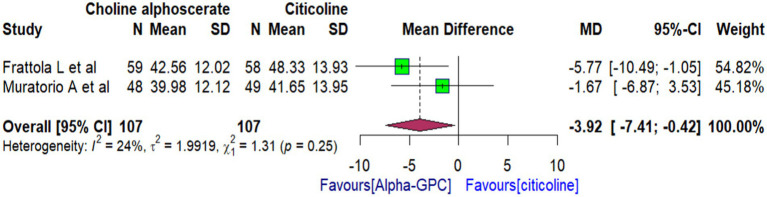
Forest plot comparing the effects of alpha-GPC versus citicoline on the clinical condition of patients with dementia disorders at the end of treatment, as measured by the SCAG global scale.

Two RCTs provided data on the overall SCAG scale scores of multi-infarct dementia (MID) patients. Based on these studies, we conducted a pooled analysis to examine the potential benefits of alpha-GPC compared to citicoline. After pooling the data from both RCTs, the findings demonstrated the positive effects of alpha-GPC on MID patients’ clinical condition compared to citicoline in the overall SCAG scale score [WMD: −3.92 (95% CI: −7.41 to −0.42)]. The analysis was associated with low heterogeneity (*I*^2^ = 24%, *p*-value = 0.25) (Figure 2).

**Figure 3:**
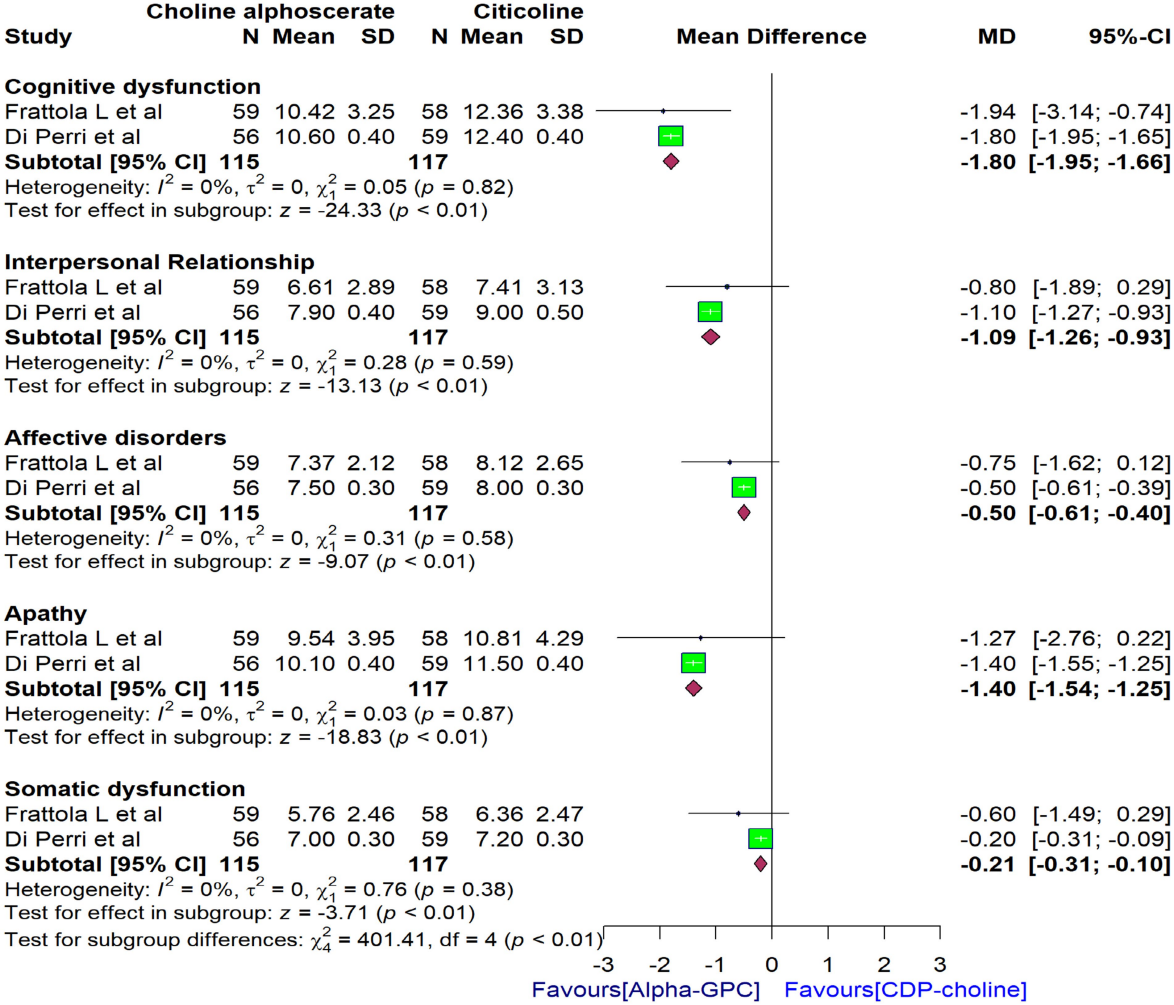
Forest plot comparing the effects of alpha-GPC versus citicoline on specific domains of the clinical condition in patients with dementia disorders at the end of treatment, as measured by the SCAG.

In addition to the findings presented above, two trials (32, 41) have reported scores for each domain of clinical condition, including cognitive dysfunction, interpersonal relationships, affective disorders, apathy, and somatic dysfunction using the SCAG scores. Accordingly, we conducted a pooled analysis to evaluate the effectiveness of alpha-GPC compared to citicoline in these specific clinical conditions. Meta-analysis revealed that alpha-GPC demonstrated significant improvements in cognitive function [WMD: −1.80 (95% CI: −1.95 to −1.66)], interpersonal relationships [WMD: −1.09 (95% CI: −1.26 to −0.93)], affective disorders [WMD: −0.50 (95% CI: −0.61 to −0.40)], apathy [WMD: −1.40 (95% CI: −1.54 to −1.25)], and somatic functioning [WMD: −0.21 (95% CI: −0.31 to −0.10)] compared to citicoline as measured by the SCAG (Figure 3).

### 3.4 Alpha-GPC versus citicoline effects on memory function and behaviour simulation

**Figure 4:**
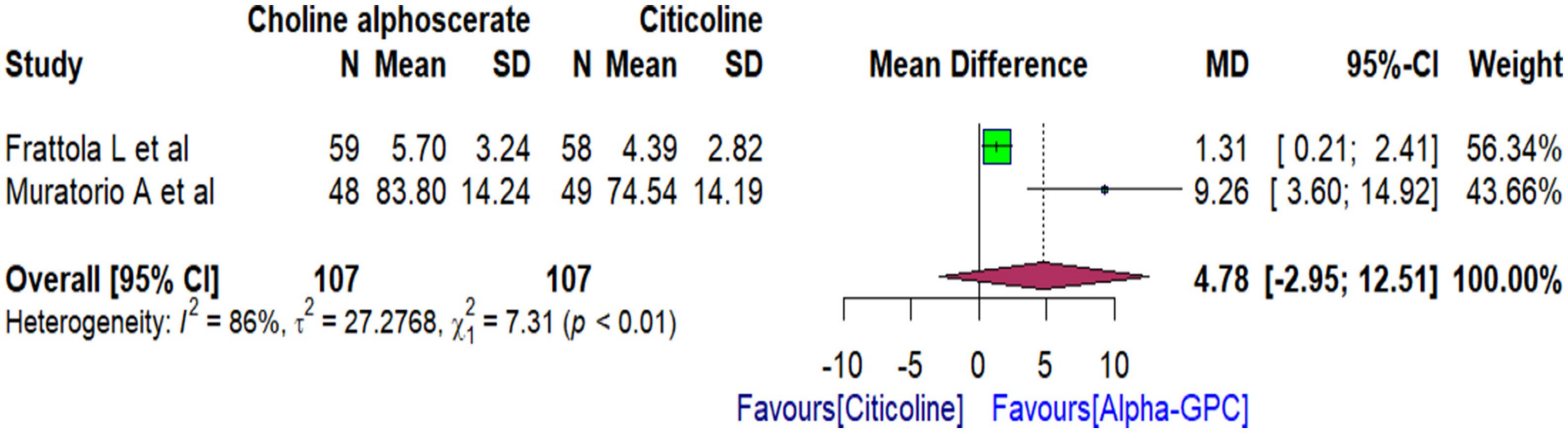
Forest plot comparing the effects of alpha-GPC versus citicoline on memory functions in MID patients at the end of treatment, as measured by WMS.

Based on findings from two RCTs (31, 41), we conducted a meta-analysis to evaluate the effectiveness of alpha-GPC versus citicoline in improving memory function and stimulated behaviour in patients with multi-infarct dementia (MID). Our analysis found that alpha-GPC did not demonstrate significant improvement compared to citicoline in improving memory functions as measured by the WMS [WMD: 4.78 (95% CI: −2.95 to 12.51)] (Figure 4).

**Figure 5:**
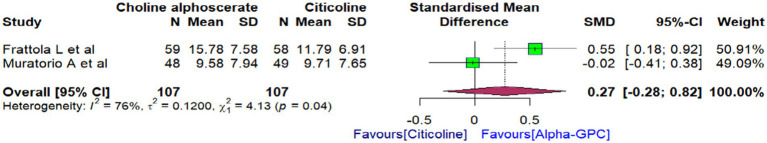
Forest plot comparing the effects of alpha-GPC versus citicoline on stimulated behaviour in MID patients at the end of treatment, as measured by MFT.

Similarly, our pooled analysis revealed no significant difference between alpha-GPC and citicoline treatments in terms of stimulated behaviour outcomes, as assessed by the WFT [SMD: 0.27; 95% CI: −0.28 to 0.82] (Figure 5). These results underscore the limited efficacy of alpha-GPC in enhancing memory function and stimulated behaviour in patients with multi-infarct dementia (MID).

### 3.5 Acceptability and tolerability

**Figure 6:**
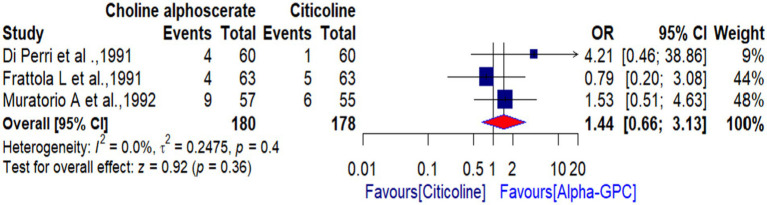
Forest plot comparing dropout rates (acceptability) between the choline alphoscerate and citicoline groups.

As for acceptability, 17 out of 180 (9.4%) patients dropped out of the studies in the alpha-GPC group, while 12 out of 178 (6.7%) patients did so in the citicoline group. However, the difference in drop-out rates between the two groups was not statistically significant [3 RCTs, OR: 1.44 (95% CI: 0.66 to 3.13)] and the between-study heterogeneity was low (*χ*^2^:1.66, *p*-value: 0.40; *I*^2^: 0.0%) (Figure 6). This indicates that both alpha-GPC and citicoline were acceptable treatment options for MID patients. In the two trials, the authors reported that dropping out (withdrawal) of the treatment was due to the occurrence of adverse effects and poor compliance with treatment in both groups. However, the remaining trial did not report the reason for the dropout of treatment. In terms of tolerability, only one out of three included trials mentioned adverse effects (AEs). Two trials did not report any AEs or did not numerically describe AEs that occurred during either alpha-GPC treatment or citicoline treatment of patients with dementia disorders. Thus, we did not perform a pooled analysis to compare the tolerability of alpha-GPC with citicoline. Nevertheless, all three included studies concluded that patients tolerated both treatments well.

### 3.6 Methodological quality of the included studies

The quality of the trials included in this systematic review was assessed using the Cochrane Collaboration tool, and the risk of bias was summarized in Supplementary Table 2. Accordingly, we assessed three included studies as having an unclear risk of bias due to their failure to adequately explain how random sequence generation was conducted. Moreover, we did not find a clear description of the allocation concealment domain; therefore, we considered the risk of bias in this domain unclear. Considering performance bias or blinding of participants or personnel, the three trials were rated as having a high risk of bias due to the lack of blinding of participants and personnel. In addition, the three studies were assessed as having a high risk of bias in terms of detection bias or outcome assessment blinding because they did not report any blinding for outcome assessment. As for the incomplete outcome data domain, we rated the risk of attrition bias as low in all three included studies. We also evaluated the risk of selective outcome reporting bias as low in the selective reporting domain because they provided appropriate information about the outcomes. Regarding other sources of bias, our rating of the three trials was unclear.

## 4 Discussion

Alpha-GPC demonstrated statistically significant improvements over citicoline in overall clinical conditions of dementia patients, as measured by the SCAG global scale score after 90 days of treatment. These results align with findings from previous randomized controlled trials, regardless of methods (20, 31, 32). Parnetti et al. (42) also found that SCAG scores were more favourable for alpha-GPC than for citicoline in patients with dementia on cognitive components. Specifically, alpha-GPC significantly improved cognitive function, interpersonal relationships, affective disorders, apathy, and somatic functioning in dementia patients compared to citicoline. Previous studies, whether compared to active treatment or placebo, showed high improvement in cognitive functions with alpha-GPC. For instance, our research group conducted a pooled data analysis to evaluate the effects of alpha-GPC versus placebo or other standard treatments on cognitive function in patients with Alzheimer’s type dementia disorders (43). The findings of this pooled data analysis provide valuable insights into the effectiveness of alpha-GPC as a treatment for cognitive impairment in individuals with dementia disorders of Alzheimer’s type (43). The observed improvement in cognitive function suggests that alpha-GPC may offer a safe and promising alternative or adjunct to existing therapeutic interventions. This finding suggests that to ensure the accuracy of results trials should conduct longer-term RCTs in this area.

Alpha-GPC provides greater and more sustained cognitive benefits than citicoline, supporting its potential role as a more effective therapeutic option for dementia and cognitive dysfunction, including early-stage cognitive decline. Different clinical studies across various populations, including those with dementia and mild cognitive impairment, consistently show that choline alphoscerate provides greater and more sustained cognitive benefits (44, 45).

In the present study, a statistically significant trend favoring alpha-GPC was observed based on the results of SCAG scores [WMD: −1.80 (95% CI: −1.95 to −1.66)], and the analysis showed low heterogeneity (*I*^2^ = 0%, *p*-value = 0.82). Our results are consistent with previously conducted reviews that have found alpha-GPC to be more effective than citicoline in enhancing cognitive function among patients with dementia (19, 46). Also, our findings align with a prior review that suggests alpha-GPC improves cognitive function in patients with vascular dementia (VaD) more effectively than citicoline and comparably or better than newer drugs (47). Alpha-GPC enhances acetylcholine (ACh) levels in the brain, which can support improving the cognitive function of patients with dementia. Different preclinical investigations suggest that alpha-GPC has multifaceted effects on the release of ACh in the rat hippocampus, cognitive function in experimental animals, brain transduction mechanisms, and age-dependent structural changes in the brain (15, 19, 48–50). Alpha-GPC is a cholinergic compound commonly used to enhance cholinergic neurotransmission. Due to its high choline content (41% by weight) and its ability to efficiently cross the blood–brain barrier, it serves as an effective choline source (51). Unlike citicoline, which undergoes various metabolic steps to release choline, alpha-GPC is directly converted into free choline upon administration (15). Consequently, it results in higher plasma choline concentrations compared to citicoline (25.8 μmol/L vs. 13.1 μmol/L) (52). These properties highlight alpha-GPC’s therapeutic potential for treating various neurological disorders.

Furthermore, our review demonstrated that alpha-GPC led to significant improvements in non-cognitive symptoms compared to citicoline, as assessed by SCAG scores. Specifically, alpha-GPC showed greater reductions in interpersonal difficulties [WMD: –1.09; 95% CI: −1.26 to −0.93], affective disturbances [WMD: –0.50; 95% CI: −0.60 to −0.40], and apathy [WMD: –1.40; 95% CI: −1.54 to −1.25]. This finding aligns with the study (41), which reported that alpha-GPC was more effective than citicoline in improving interpersonal relationships in patients with multi-infarct dementia (MID). Different randomized controlled trials (RCTs) involving patients with Alzheimer’s disease have shown that the combination of alpha-GPC and donepezil significantly reduces symptoms of depression and apathy compared to treatment with donepezil alone (53–55). Additionally, a study conducted in healthy volunteers found that alpha-GPC significantly enhanced motivation when compared to a placebo group (56). Alpha-GPC’s primary effects on cognitive function may indirectly benefit interpersonal relationships by enhancing mental clarity, reducing cognitive deficits, and supporting better communication and social interactions. Alpha-GPC appears to significantly reduce affective disturbances and apathy, which may be linked to its ability to alleviate symptoms of anxiety and depression associated with affective disorders. These effects are potentially due to its influence on brain neurotransmitter levels and overall neural health. Moreover, alpha-GPC has been shown to reduce apathy in patients with dementia, possibly by enhancing motivation and cognitive engagement. Its modulation of dopamine and serotonin levels, neurotransmitters critical for mood regulation and emotional processing, may underlie these benefits.

We found that alpha-GPC is more effective than citicoline in improving somatic functioning, as measured by the SCAG [WMD: −0.21 (95% CI: −0.31 to −0.10)] at the end of treatment. Our finding agrees with previously conducted RCTs (41), which found that alpha-GPC significantly improved the somatic functioning of patients with dementia at the end of treatment. Alpha-GPC may offer neuroprotective benefits and improve functional outcomes in neurological conditions due to its potential anti-inflammatory properties, which support overall brain health and function (57).

The dropout rates for both treatments were nearly similar, with no significant difference between the two groups [OR: 1.44 (95% CI: 0.66 to 3.13)], suggesting that both treatments were acceptable for dementia patients. Although a meta-analysis for tolerability was not conducted due to insufficient data, the included studies indicated that both choline alphoscerate and citicoline were well tolerated in dementia patients.

### 4.1 Limitations of this study

The present systematic review evaluates the most effective interventions for dementia disorders through RCTs, but it is limited by the small number of included studies and their low methodological quality. As another limitation, the studies included in our systematic review were old in terms of publication date, even though they were scientifically relevant. However, their scientific relevance indicates that these studies continue to contribute valuable insights and foundational knowledge to the field. Despite their age, the included studies provide a valuable baseline for new research and assist in understanding long-term trends. This ongoing relevance underscores the importance of including such studies in our review to provide a comprehensive overview. Moreover, our analysis was limited to articles written in English, potentially excluding valuable data published in other languages, which may affect the generalizability of the findings. Another limitation was the inability to assess publication bias due to the limited number of included studies in the meta-analysis (34, 58). Finally, our review is limited to vascular dementia or multi-infarct dementia types, so the results cannot be directly generalized to other forms of dementia, such as Alzheimer’s disease and other non-vascular types.

## 5 Conclusion

In summary, this systematic review and meta-analysis demonstrated that alpha-GPC significantly improved overall clinical outcomes in patients with dementia, with notable benefits in cognitive function, interpersonal relationships, affective symptoms, apathy, and somatic health, compared to citicoline. These results highlight alpha-GPC’s potential as an effective therapeutic option for individuals with neurological disorders, particularly dementia. By boosting acetylcholine production, enhancing neuroprotection, promoting synaptic plasticity, and regulating emotional and behavioural responses, alpha-GPC could offer significant benefits to those experiencing cognitive impairments. However, due to methodological limitations, including open-label designs, short treatment duration, and small sample sizes, the strength of this conclusion remains limited. Future large-scale, double-blind randomized controlled trials with extended follow-up and broader outcome measures are necessary to confirm these results.

## Data Availability

The original contributions presented in the study are included in the article/Supplementary material, further inquiries can be directed to the corresponding author.

## References

[1] Nilaweera D Gurvich C Freak-Poli R Woods R Owen A Murray A . Adverse events in older adults and the risk of dementia and cognitive decline. J Affect Disord Rep. (2023) 13:100592. doi: 10.1016/j.jadr.2023.100592, PMID: 37475782 PMC10357969

[2] McKhann GM Knopman DS Chertkow H Hyman BT Jack CR Jr Kawas CH . The diagnosis of dementia due to Alzheimer’s disease: recommendations from the National Institute on Aging-Alzheimer’s Association workgroups on diagnostic guidelines for Alzheimer’s disease. Alzheimers Dement. (2011) 7:263–9. doi: 10.1016/j.jalz.2011.03.005, PMID: 21514250 PMC3312024

[3] Chin KS. Pathophysiology of dementia. Aust J Gen Pract. (2023) 52:516–21. doi: 10.31128/AJGP-02-23-6736, PMID: 37532448

[4] Prince M Guerchet M Prina M. The global impact of dementia 2013–2050. Lincolnshire, IL: Alzheimer’s Disease International (2013).

[5] Wimo A Jönsson L Bond J Prince M Winblad B Alzheimer Disease International. The worldwide economic impact of dementia 2010. Alzheimers Dement. (2013) 9:1. doi: 10.1016/j.jalz.2012.11.006, PMID: 23305821

[6] Langa KM. Is the risk of Alzheimer’s disease and dementia declining? Alzheimers Res Ther. (2015) 7:34. doi: 10.1186/s13195-015-0118-1, PMID: 25815064 PMC4374373

[7] Ritchie CW Terrera GM Quinn TJ. Dementia trials and dementia tribulations: methodological and analytical challenges in dementia research. Alzheimers Res Ther. (2015) 7:31. doi: 10.1186/s13195-015-0113-6, PMID: 25788988 PMC4364079

[8] Takramah WK Asem L. The efficacy of pharmacological interventions to improve cognitive and behavior symptoms in people with dementia: a systematic review and meta-analysis. Health Sci Rep. (2022) 5:e913. doi: 10.1002/hsr2.913, PMID: 36381407 PMC9637987

[9] Tricco AC Soobiah C Berliner S Ho JM Ng CH Ashoor HM . Efficacy and safety of cognitive enhancers for patients with mild cognitive impairment: a systematic review and meta-analysis. CMAJ. (2013) 185:1393–401. doi: 10.1503/cmaj.130451, PMID: 24043661 PMC3826344

[10] Cooper C Li R Lyketsos C Livingston G. Treatment for mild cognitive impairment: systematic review. Br J Psychiatry. (2013) 203:255–64. doi: 10.1192/bjp.bp.113.127811, PMID: 24085737 PMC3943830

[11] Feldman HH Ferris S Winblad B Sfikas N Mancione L He Y . Effect of rivastigmine on delay to diagnosis of Alzheimer’s disease from mild cognitive impairment: the InDDEx study. Lancet Neurol. (2007) 6:501–12. doi: 10.1016/S1474-4422(07)70109-6, PMID: 17509485

[12] Thal LJ Ferris SH Kirby L Block GA Lines CR Yuen E . A randomized, double-blind, study of rofecoxib in patients with mild cognitive impairment. Neuropsychopharmacology. (2005) 30:1204–15. doi: 10.1038/sj.npp.1300690, PMID: 15742005

[13] Schneider SL. A critical review of cholinesterase inhibitors as a treatment modality in Alzheimer’s disease. Dialogues Clin Neurosci. (2000) 2:111–28. doi: 10.31887/DCNS.2000.2.2/lschneider 22033801 PMC3181592

[14] Hogan DB. Long-term efficacy and toxicity of cholinesterase inhibitors in the treatment of Alzheimer disease. Can J Psychiatry. (2014) 59:618–23. doi: 10.1177/070674371405901202, PMID: 25702360 PMC4304580

[15] Traini E Bramanti V Amenta F. Choline alphoscerate (alpha-glyceryl-phosphoryl-choline) an old choline- containing phospholipid with a still interesting profile as cognition enhancing agent. Curr Alzheimer Res. (2013) 10:1070–9. doi: 10.2174/15672050113106660173, PMID: 24156263

[16] Rosenberg G Davis K. The use of cholinergic precursors in neuropsychiatric diseases. Am J Clin Nutr. (1982) 36:709–20. doi: 10.1093/ajcn/36.4.709, PMID: 6214943

[17] De Jesus Moreno Moreno M. Cognitive improvement in mild to moderate Alzheimer’s dementia after treatment with the acetylcholine precursor choline alfoscerate: a multicenter, double-blind, randomized, placebo-controlled trial. Clin Ther. (2003) 25:178–93. doi: 10.1016/s0149-2918(03)90023-3, PMID: 12637119

[18] Premi E Cantoni V Benussi A Gilberti N Vergani V Delrio I . Citicoline treatment in acute ischemic stroke: a randomized, single-blind TMS study. Front Neurol. (2022) 13:915362. doi: 10.3389/fneur.2022.915362, PMID: 35923827 PMC9340348

[19] Parnetti L Mignini F Tomassoni D Traini E Amenta F. Cholinergic precursors in the treatment of cognitive impairment of vascular origin: ineffective approaches or need for re-evaluation? J Neurol Sci. (2007) 257:264–9. doi: 10.1016/j.jns.2007.01.043, PMID: 17331541

[20] Parnetti L Abate G Bartorelli L Cucinotta D Cuzzupoli M Maggioni M . Multicentre study of l-alpha-glyceryl-phosphorylcholine vs ST200 among patients with probable senile dementia of Alzheimer’s type. Drugs Aging. (1993) 3:159–64. doi: 10.2165/00002512-199303020-00006, PMID: 8477148

[21] Sangiorgi GB Barbagallo M Giordano M Meli MARIA Panzarasa RITA. alpha-Glycerophosphocholine in the mental recovery of cerebral ischemic attacks. An Italian multicenter clinical trial. Ann N Y Acad Sci. (1994) 717:253–69. doi: 10.1111/j.1749-6632.1994.tb12095.x, PMID: 8030842

[22] Buĭlova TV Glotova ME Khalak ME Vashkevich VV. The use of cereton in the rehabilitation of patients with hemorrhagic stroke. Zh Nevrol Psikhiatr Im S S Korsakova. (2009) 109:57–61. PMID: 19894302

[23] Secades JJ Lorenzo JL. Citicoline: pharmacological and clinical review, 2006 update. Methods Find Exp Clin Pharmacol. (2006) 28:1–56. 17171187

[24] Gareri P Castagna A Cotroneo AM Putignano S de Sarro G Bruni AC. The role of citicoline in cognitive impairment: pharmacological characteristics, possible advantages, and doubts for an old drug with new perspectives. Clin Interv Aging. (2015) 10:1421. doi: 10.2147/CIA.S87886, PMID: 26366063 PMC4562749

[25] Fioravanti M Yanagi M. Cytidinediphosphocholine (CDP choline) for cognitive and behavioural disturbances associated with chronic cerebral disorders in the elderly. Cochrane Database Syst Rev. (2004) 2:CD000269. doi: 10.1002/14651858.CD000269.pub2 15106147

[26] Caamaño J Gómez MJ Franco A Cacabelos R. Effects of CDP-choline on cognition and cerebral hemodynamics in patients with Alzheimer’s disease. Methods Find Exp Clin Pharmacol. (1994) 16:211–8. 7913981

[27] Fernández-Novoa L Alvarez XA Franco-Maside A Caamaño J Cacabelos R. CDP-choline-induced blood histamine changes in Alzheimer’s disease. Methods Find Exp Clin Pharmacol. (1994) 16:279–84. 8051988

[28] Cacabelos R Caamaño J Gómez MJ Fernández‐Novoa L Franco‐Maside A Alvarez XA. Therapeutic effects of CDP-choline in Alzheimer’s disease-cognition, brain mapping, cerebrovascular hemodynamics, and immune factors. Ann N Y Acad Sci. (1996) 777:399–403. doi: 10.1111/j.1749-6632.1996.tb34452.x, PMID: 8624120

[29] Alvarez XA Mouzo R Pichel V Pérez P Laredo M Fernández-Novoa L . Double-blind placebo-controlled study with citicoline in APOE genotyped Alzheimer’s disease patients. Effects on cognitive performance, brain bioelectrical activity and cerebral perfusion. Methods Find Exp Clin Pharmacol. (1999) 21:633–44. doi: 10.1358/mf.1999.21.9.795632, PMID: 10669911

[30] Zafonte RD Bagiella E Ansel BM Novack TA Friedewald WT Hesdorffer DC . Effect of Citicoline on functional and cognitive status among patients with traumatic brain injury. JAMA. (2012) 308:1993–2000. doi: 10.1001/jama.2012.13256, PMID: 23168823

[31] Muratorio A Bonuccelli U Nuti A Battistini N Passero S Caruso V . A neurotropic approach to the treatment of multi-infarct dementia using L-α-glycerylphosphorylcholine. Curr Ther Res. (1992) 52:741–52. doi: 10.1016/S0011-393X(05)80518-1

[32] Di Perri R Coppola G Ambrosio LA Grasso A Puca FM Rizzo M. A multicentre trial to evaluate the efficacy and tolerability of α-glycerylphosphorylcholine versus cytosine diphosphocholine in patients with vascular dementia. J Int Med Res. (1991) 19:330–41. doi: 10.1177/030006059101900406, PMID: 1916007

[33] Moher D Liberati A Tetzlaff J Altman DG The PRISMA Group. Preferred reporting items for systematic reviews and meta-analyses: the PRISMA statement. PLoS Med. (2009) 6:e1000097. doi: 10.1371/journal.pmed.1000097, PMID: 19621072 PMC2707599

[34] Higgins JP. Cochrane handbook for systematic reviews of interventions. Hoboken, NJ: Wiley-Blackwell (2020).

[35] R Core Team. R: A language and environment for statistical, computing. Vienna: Foundation for Statistical Computing (2019).

[36] Viechtbauer W. Conducting meta-analyses in {R} with the {metafor} package. J Stat Softw. (2010) 36:1–48. doi: 10.18637/jss.v036.i03

[37] Sasse EC Sasse AD Brandalise SR Clark OAC Richards S Cochrane Haematological Malignancies Group. Colony-stimulating factors for prevention of myelosuppressive therapy-induced febrile neutropenia in children with acute lymphoblastic leukaemia. Cochrane Database Syst Rev. (2005) 3:CD004139. doi: 10.1002/14651858.CD004139.pub2, PMID: 16034921 PMC12935170

[38] Tufanaru C Munn Z Stephenson M Aromataris E. Fixed or random effects meta-analysis? Common methodological issues in systematic reviews of effectiveness. Int J Evid Based Healthc. (2015) 13:196–207. doi: 10.1097/XEB.0000000000000065, PMID: 26355603

[39] Edwards AG Naik G Ahmed H Elwyn GJ Pickles T Hood K . Personalised risk communication for informed decision making about taking screening tests. Cochrane Database Syst Rev. (2013) 2013:CD001865. doi: 10.1002/14651858.CD001865.pub3 23450534 PMC6464864

[40] DerSimonian R Laird N. Meta-analysis in clinical trials. Control Clin Trials. (1986) 7:177–88. doi: 10.1016/0197-2456(86)90046-2, PMID: 3802833

[41] Frattola L Piolti R Bassi S Albizzati MG. Multicenter clinical comparison of the effects of choline alfoscerate and cytidine diphosphocholine in the treatment of multi-infarct dementia. Curr Ther Res. (1991) 49:683–93.

[42] Parnetti L Amenta F Gallai V. Choline alphoscerate in cognitive decline and in acute cerebrovascular disease: an analysis of published clinical data. Mech Ageing Dev. (2001) 122:2041–55. doi: 10.1016/S0047-6374(01)00312-8, PMID: 11589921

[43] Sagaro GG Traini E Amenta F. Activity of choline alphoscerate on adult-onset cognitive dysfunctions: a systematic review and meta-analysis. J Alzheimers Dis. (2023) 92:59–70. doi: 10.3233/JAD-221189, PMID: 36683513 PMC10041421

[44] Jeon J Lee SY Lee S Han C Park GD Kim SJ . Efficacy and safety of choline alphoscerate for amnestic mild cognitive impairment: a randomized double-blind placebo-controlled trial. BMC Geriatr. (2024) 24:774. doi: 10.1186/s12877-024-05366-7, PMID: 39300341 PMC11412009

[45] Ponomareva EV Androsova LV Krinsky SA Gavrilova SI. Efficacy and safety of choline alfoscerate in the preventive therapy of dementia in elderly patients with mild cognitive impairment: a three-year prospective comparative study. Zh Nevrol Psikhiatr Im S S Korsakova. (2024) 124:92. doi: 10.17116/jnevro202412404292, PMID: 38696157

[46] Biggio G Mencacci C. Choline alphoscerate: insights between acquired certainties and future perspectives. Front Aging Neurosci. (2025) 17:1613566. doi: 10.3389/fnagi.2025.1613566, PMID: 40842650 PMC12364881

[47] Amenta F Di Tullio MA Tomassoni D. The cholinergic approach for the treatment of vascular dementia: evidence from pre-clinical and clinical studies. Clin Exp Hypertens. (2002) 24:697–713. doi: 10.1081/CEH-120015346, PMID: 12450245

[48] Lopez CM Govoni S Battaini F Bergamaschi S Longoni A Giaroni C . Effect of a new cognition enhancer, alpha-glycerylphosphorylcholine, on scopolamine-induced amnesia and brain acetylcholine. Pharmacol Biochem Behav. (1991) 39:835–40. doi: 10.1016/0091-3057(91)90040-9, PMID: 1662399

[49] Amenta F Franch F Ricci A Vega JA. Cholinergic neurotransmission in the hippocampus of aged rats: influence of L-α-glycerylphosphorylcholine treatment. Ann N Y Acad Sci. (1993) 695:311–3. doi: 10.1111/j.1749-6632.1993.tb23073.x, PMID: 8239302

[50] Sigala S Imperato A Rizzonelli P Casolini P Missale C Spano PF. L-α-glycerylphorylcholine antagonizes scopolamine-induced amnesia and enhances hippocampal cholinergic transmission in the rat. Eur J Pharmacol. (1992) 211:351–8. doi: 10.1016/0014-2999(92)90392-H 1319912

[51] Kansakar U Trimarco V Mone P Varzideh F Lombardi A Santulli G. Choline supplements: an update. Front Endocrinol. (2023) 14:1148166. doi: 10.3389/fendo.2023.1148166, PMID: 36950691 PMC10025538

[52] Gatti G Barzaghi N Acuto G Abbiati G Fossati T Perucca E. A comparative study of free plasma choline levels following intramuscular administration of L-alpha-glycerylphosphorylcholine and citicoline in normal volunteers. Int J Clin Pharmacol. (1992) 30:331–5. 1428296

[53] Rea R Carotenuto A Traini E Fasanaro AM Manzo V Amenta F. Apathy treatment in Alzheimer’s disease: interim results of the ASCOMALVA trial. J Alzheimers Dis. (2015) 48:377–83. doi: 10.3233/JAD-141983, PMID: 26402001

[54] Carotenuto A Rea R Traini E Fasanaro AM Ricci G Manzo V . The effect of the association between donepezil and choline alphoscerate on behavioral disturbances in Alzheimer’s disease: interim results of the ASCOMALVA trial. J Alzheimers Dis. (2017) 56:805–15. doi: 10.3233/JAD-160675, PMID: 28035924

[55] Carotenuto A Fasanaro AM Manzo V Amenta F Traini E. Association between the cholinesterase inhibitor donepezil and the cholinergic precursor choline alphoscerate in the treatment of depression in patients with Alzheimer’s disease. J Alzheimers Dis Rep. (2022) 6:235–43. doi: 10.3233/ADR-200269, PMID: 35719710 PMC9198805

[56] Tamura Y Takata K Matsubara K Kataoka Y. Alpha-glycerylphosphorylcholine increases motivation in healthy volunteers: a single-blind, randomized, placebo-controlled human study. Nutrients. (2021) 13:2091. doi: 10.3390/nu13062091, PMID: 34207484 PMC8235064

[57] Roy P Tomassoni D Nittari G Traini E Amenta F. Effects of choline containing phospholipids on the neurovascular unit: a review. Front Cell Neurosci. (2022) 16:988759. doi: 10.3389/fncel.2022.988759, PMID: 36212684 PMC9541750

[58] Egger M Smith GD Schneider M Minder C. Bias in meta-analysis detected by a simple, graphical test. BMJ. (1997) 315:629–34. doi: 10.1136/bmj.315.7109.629, PMID: 9310563 PMC2127453

