# Supplementary material for: Comparison of the effects of choline alphoscerate and citicoline in patients with dementia disorders: a systematic review and meta-analysis
Source: Front Neurol. 2025 Dec 5;16:1649661. doi: 10.3389/fneur.2025.1649661 (PMC12714635; doi:10.3389/fneur.2025.1649661)
Supplement: Supplementary file 1 [file Table_1.docx]

**Supplemental Data**

Comparison of the Effects of Choline Alphoscerate and Citicoline in Patients with Dementia Disorders: A Systematic Review and Meta-analysis

*Getu Gamo* ***Sagaro****^1,2^ and Francesco* ***Amenta****^1,3^*

**TABLE OF CONTENTS**

1. Table 1: Search strategy
2. Table 2: Appraisal of the included randomized controlled trials (RCT) risk of bias using the Cochrane risk of bias tool.

**Supplemental Table 1: Search strategy**

1. Citicoline OR cytidine diphosphate choline OR CDP-choline OR cytidine 5’-diphosphocholine OR citocholine OR cyticholine OR cytidine-5-diphosphocholine
2. L-Alpha glycerylphosphorylcholine OR alpha-GPC OR choline alphoscerate OR ceremony OR α-Glyceryphosphorylcholine OR alpha- glyceryl-phosphorylcholine OR α-GPC
3. dementia OR dementia diseases OR dementia disorders OR Alzheimer's disease OR Alzheimer's/AD OR vascular dementia OR frontotemporal dementia OR mixed dementia OR cognitive impairment OR multi-infarct dementia/MID OR multiinfarct dementia.


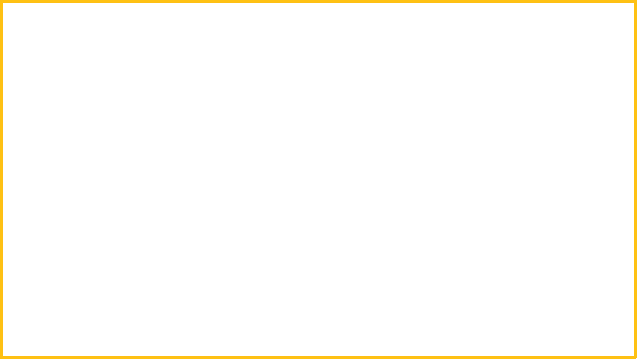


1. 1 AND 2
2. 4 AND 3
3. Limit 5 to humans
4. Omit duplicates from 6

**Supplementary Table 2**. Appraisal of risk of bias of the included randomized controlled trials (RCTs) using the Cochrane risk of bias tool

| Trial | Random sequence generation | Allocation concealment | Blinding of participants, personnel | Blinding of outcome assessors | Incomplete outcome data | Selective outcome reporting | Other sources of bias | Classification |
| --- | --- | --- | --- | --- | --- | --- | --- | --- |
| Frattola L et al.,1991 | Unclear | Unclear | No | No | Yes | Yes | Unclear | High |
| Muratorio A et al.,1992 | Unclear | Unclear | No | No | Yes | Yes | Unclear | High |
| Di Perri et al.,1991 | Unclear | Unclear | No | No | Yes | Yes | Unclear | High |
